# A Simple and Cost-Effective Freeze-Thaw Based Method for *Plasmodium* DNA Extraction from Dried Blood Spot

**Published:** 2019

**Authors:** Supriya SHARMA, Riti MANN, Sandeep KUMAR, Neelima MISHRA, Bina SRIVASTAVA, Neena VALECHA, Anupkumar R. ANVIKAR

**Affiliations:** ICMR-National Institute of Malaria Research, Sector 8, Dwarka, New Delhi, 110077, India

**Keywords:** *Plasmodium*, DNA isolation, Freezing, Thawing, PCR

## Abstract

**Background:**

Available DNA isolation methods for *Plasmodium* involve numerous processing steps, adding to the cost and conferring risk of contamination. Here we devise a simple and cost-effective method for direct extraction of *Plasmodium* DNA from dried filter paper spot (DBS), appropriate for resource-limited setups.

**Methods:**

The protocol involves simple freezing and thawing of DBS, neither involves any purification step nor any chemical reagent. The method was assessed in terms of DNA quantity, PCR detection sensitivity, time requirement, cost effectiveness, labor intensiveness and degree of shearing. The reliability of this method was confirmed by comparing it with other in use methods for *Plasmodium* DNA isolation.

**Results:**

Pure DNA was obtained with this method, as exemplified by the absorbance ratio (260nm /280nm) of 1.2. The protocol produced digestible, PCR-grade genomic DNA, also found to be suitable for sequencing. DNA isolated remained stable and retained its integrity after storage for one month at 4 °C.

**Conclusion:**

Our process substantiated as efficient, reproducible, simple, fast, and inexpensive. Development of this optimized freeze-thaw based DNA extraction method for malaria parasite may provide a valuable tool for molecular analysis in resource-limited setups. This is the first report of DNA extraction from DBS of *Plasmodium* utilizing freeze-thaw.

## Introduction

Malaria caused by *Plasmodium* sp. is a serious global health problem, leading to about 584,000 fatalities annually (1). Apt and correct diagnosis is necessary for prevention, control and suitable treatment of malaria. Accurate diagnosis cannot be achieved by microscopy or Rapid Diagnostic Tests (RDTs) alone, as they both have certain shortcomings. Molecular methods being sensitive and specific, capable of detecting submicroscopic and mixed infections are best suited for confirmatory diagnosis of malaria (2). Success of a molecular method for malaria diagnosis depends majorly on the quality of genomic DNA being used for examination, which in turn depends on the efficacy of employed isolation procedure. Numerous techniques available for isolating *Plasmodium* DNA can broadly be classified into chemical-based methods, matrix-based methods and the use of commercial kits (3). Besides these, approaches like irradiation (4) and isotachophoresis (5) have also been used for malaria parasite DNA isolation. These presently available protocols are either very expensive, laborious, require expertise, or involve a number of chemical processing steps. Due to these drawbacks, they often fail to demonstrate their applicability in field setups (3).

Chemical techniques involve the use of enzymes such as lysozyme and proteinase K, and detergents like sodium dodecyl sulfate (SDS) and cetyltrimethylammonium bromide (CTAB) for the cell lysis which is first and most critical stage in the DNA isolation. These chemicals dissolve and disrupt the cell membranes, allowing DNA and proteins to come out of the cells (6). Under physical methods of cell lysis techniques like sonication, mechanical disruption, liquid homogenization, manual grinding and freeze-thaw can be used. Applying bead beating as a physical approach for DNA isolation shears the DNA (7). Therefore, gentle lysis methods such as freeze-thawing treatments are better for getting intact DNA. Johnson and Hecht 1994 reported the use of freezing and thawing for releasing recombinant proteins from cells. Rapid freezing can be accomplished in dry ice with methanol or ethanol (8, 9) or by incubation in liquid nitrogen. For slow freezing samples are frozen at low temperatures, usually in the range −20 °C to −80 °C. Freeze-thaw based approach has been attempted for DNA isolation from various sources like dental plaque. Giemsa-stained bone marrow slides, soil and sediment samples (7), microbial mats, activated sludge (10), and from organisms such as *Mycobacterium avium*, *Echinococcus granulosus*, *Giardia lambli*, *Giardia intestinalis*, *Salmonella*, yeasts, *Botryococcusbraunii,* and *Giardia duodenalis*.

DNA extraction protocols employing freeze-thaw as a physical method for cell disruption often simultaneously involve the use of chemicals (8) and many times are followed by further purification steps. These protocols apply multiple freeze-thaw cycles for cell lysis (8, 9, 11–14) which ultimately affect the DNA integrity leading to production of small DNA fragments. Long term and repeated freezing-thawing of samples enhance cell lysis (15), but concurrently also increase the processing steps and chances of contamination. Repeated thawing also reduces the sensitivity of detection techniques such as PCR by as greatly as 10 times (2). Considering all these factors the purpose of this study was to develop a simple, cost-effective and less labor-intensive isolation method for *Plasmodium*.

## Materials and Methods

### Samples

*P. falciparum* dried blood spots of field samples (IN002-31, IN002-37, and ML-6) and culture adapted samples (NF-54 and RKL-9) were used in the study; mentioned hereafter as FP1, FP2, FP3, FP4, and FP5 respectively. Parasitemia and parasites/μl for each sample are mentioned in Table 1. MRC-2 and RKL-9, chloroquine sensitive and resistant strain respectively, were used as standard laboratory isolates, from Malaria Parasite Bank of National Institute of Malaria Research (NIMR), New Delhi.

**Table 1:** Sample Id and detail of parasitemia of samples used in this study

***Sample***	***Sample Id***	***Parasitemia***	***Parasites/μl***
IN002-31	FP 1	~ 3 %	~1,50,000
IN002-37	FP 2	~3.5%	~1,75,000
ML-6	FP 3	~0.1%	~5,000
NF-54	FP 4	~2%	~1,00,000
RKL-9	FP 5	~1%	~50,000

### Ethics Statement

This work has been approved by Ethical Committee of ICMR-NIMR, New Delhi.

### Freeze-thaw based DNA extraction

The dried blood spot was prepared using 25μl of parasitized blood and spreading it in circular manner. It was allowed to dry for 8 h at room temperature. Six punches of DBS (3mm in diameter) were transferred to six separates 1.5 ml microtubes. Sterile distilled water (200 μl) was added to three of these tubes and 200 μl of phosphate buffered saline (PBS) to the remaining tubes. One tube out of these two sets was incubated at following temperatures: 0 °C, −20 °C, −80 °C for overnight. After incubation, the samples were taken out and thawed at 37 °C for 15–30 min, vortexed for few seconds and centrifuged at 8000 rpm for two min.

**Fig. 1:**
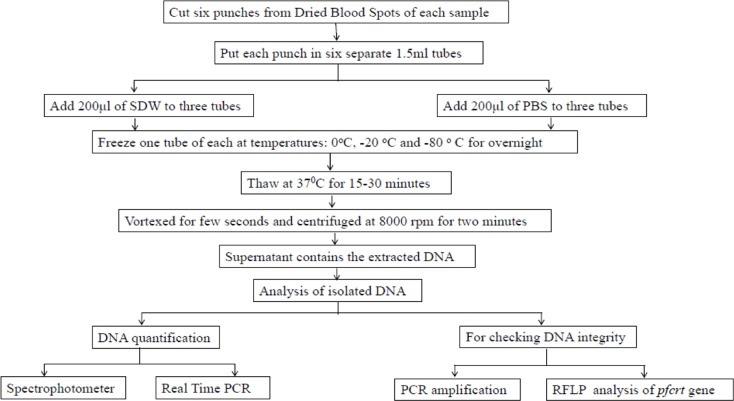
Experimental Workflow: the process of DNA extraction from Dried Blood spots using freeze-thaw method and methodology followed for analysis of isolated DNA

The resultant supernatant contained the isolated DNA. Figure 1 shows the experimental Workflow of DNA extraction process from Dried Blood spots using freeze-thaw method and various methodologies followed for analysis of isolated DNA.

### DNA Quantification using Real-Time PCR

DNA isolated using freeze-thaw based approach was quantified spectrophotometrically using Nanodrop Q-5000 UV-Vis Spectrophotometer (San Jose, CA, USA) and by absolute quantification using Real-Time PCR. DNA purity was evaluated by comparing the absorbance ratios at 260 nm to 280 nm. Dilution curves of all samples were analyzed by real-time PCR and against known (given by the Nanodrop Spectrophotometer) DNA concentrations the Ct-values were plotted.

The standard DNA for quantification using Real-Time PCR was prepared using 3D7 strain by Psp1 and Psp2 primers amplification. The amplified sequence was inserted into plasmid pGEMT-Easy vector (Promega, Madison, WI, USA) to generate recombinant plasmid. This recombinant plasmid was transformed into *Escherichia coli* DH5α cells (NEB, UK). Positive clone was screened through colony PCR and the selected recombinant plasmid was isolated using MDI Plasmid isolation kit (MDI, India). *Plasmodium* DNA standard was measured with spectrophotometer and prepared by serial dilutions in sterile distilled water, ranging from 5×10^7^ to 5×10^2^ plasmid copies per μl. Standard curves were generated in each experiment from real-time quantification of standard DNA dilutions. Sample DNA loads were calculated using the Light Cycler 480 (Roche) analysis software. The threshold cycle (Ct), defined as the fractional cycle at which the fluorescence signal becomes significantly different from the baseline signal, was determined for each sample. The unknown sample DNA loads were calculated from their Ct values and compared with the standard curve.

### DNA Quantification using PCR

To evaluate DNA quantity and sensitivity with dried blood spot from different numbers of parasites (10, 100, 1000, 10000…1000000 parasites/spot) with freeze-thaw samples, we diluted culture sample (FF4) of parasitemia 100000 parasite /μl to 100 parasite /μl,10 parasite /μl, 1 parasite /μl, 0.1 parasite /μl in PBS. The mitochondrial gene was amplified for these diluted sample using single-step PCR (ABI,USA).

### PCR Amplification

Amplifications targeting diverse genetic fragments were performed for confirming the identity of isolated *Plasmodium* DNA. All these amplifications were carried out in a final reaction volume of 20μl using Thermal cycler (ABI, USA). Each of the reaction mixtures consisted of 10μl Thermo Scientific Dream Taq Green PCR Master Mix (2X), 1μl forward primer, 1μl reverse primer, 6μl sterile distilled water and 2 μl of template DNA. PCR reactions for diagnosis of malaria parasites were performed targeting two different genes: *Plasmodium mitochondrial gene* (*mt gene*) and nested PCR assay initially targeting *18S rRNA gene* followed by amplification using *P. falciparum* species-specific primers (16). Amplifications of single copy genes *msp1* and *msp2* were done for testing the integrity of isolated DNA.

### Restriction Fragment Length Polymorphism (RFLP) Analysis

The digestibility of isolated DNA was assessed by RFLP analysis of *pfcrt gene* using ApoI restriction enzyme. 4μl of PCR amplified product of *pfcrt gene* was digested using 1U of ApoI enzyme (NEB, UK) by incubation in buffer at 37 °C for 2 h.

### Electrophoresis

The PCR-amplified products (10μl) and 100 bp DNA ladder (GeNei, USA) were loaded onto 2% agarose (Merck, USA) electrophoresis gel containing 0.5 μg/ml Etbr (GeNei, USA). ApoI digested product was analyzed on 2.5% agarose gel. The run was performed in 0.5 X TBE buffer (pH 8.0) for 40 min. The gels were visualized under UV light and the image captured using gel documentation system (Alpha Innotech, USA).

### DNA Sequencing

The authenticity of isolated DNA was checked by sequence analysis of *msp1* and *msp2* gene alleles. PCR amplified products were sequenced for each specific allele. The PCR products were sent to Xcelris Labs, Ahmedabad, Gujarat, India for purification and DNA sequencing. Each sample was sequenced with both forward and reverse primers. The editing and alignment of DNA sequences were done using Bio Edit Sequence Alignment Editor Software.

### Comparison with other DNA extraction methods

Freeze-thaw based DNA extraction method was compared with other DNA extraction methods: chemical-based methods-Tris-EDTA and Saponin-EDTA (17), physical extraction method- microwave irradiation (4), and commercially available kits-Qiagen QI Amp Blood Extraction Kit (Qiagen, Germany) and Genomic DNA Mini Kit (B R Biochem, India).

### DNA stability

DNA samples prepared in PBS at 0 °C were stored at 4 °C and ambient temperature (28 °C) for 30 d. PCR amplification was performed for mitochondrial genes at 1st, 2nd, 3rd, 7th and 30th day after storage, checked DNA stability at these two storing temperatures.

## Results

The current study appraised the applicability of freezing and thawing as a process to isolate *Plasmodium* DNA from DBS samples using SDW and PBS as final eluting solution. Five samples, all in triplicates at three different freezing temperatures were considered for analysis.

### DNA concentration

**Fig. 2:**
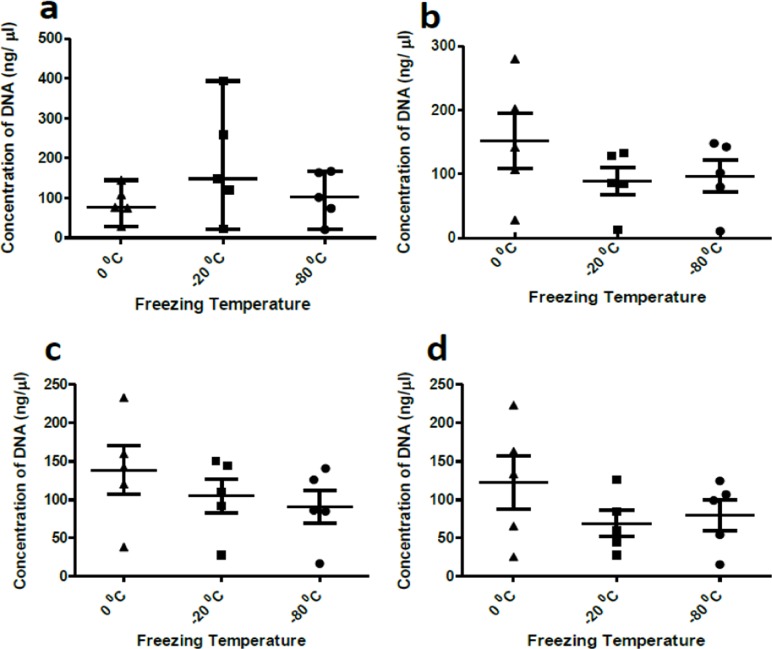
Error bars representing the mean standard errors of DNA concentrations for three different temperatures (0 °C, −20 °C, −80 °C) (a) DNA concentration in SDW on zero-day (b) DNA concentration in SDW on 30th day after storage at 0 °C (c) DNA concentration in PBS on zero-day (d) DNA concentration in PBS on 30th day after storage at 0 °C

The isolated DNA was quantified using spectrophotometer immediately after freezing-thawing process and after storage at 0 °C for 30 d. Mean DNA concentrations at zero-day using SDW as the eluting solution was found to be 86.7 ng/μl for freezing temperature of 0 °C, 188.7 ng/μl for −20 °C and 105.3 ng/μl for −80 °C. After 30 d the concentrations were 151.78 ng/μl, 88.84 ng/μl and 96.5 ng/μl respectively. DNA concentration values were slightly lower in case of PBS with values at zero-day being 139.02 ng/μl for 0 °C, 105.10 ng/μl for −20 °C and 91.08 ng/μl for −80 °C. After 30 d of DNA storage, these concentrations reduced to 122.44 ng/μl for 0 °C, 69.04 ng/μl for −20 °C and 80.22 ng/μl for −80 °C (Fig. 2).

### DNA Quantification using Real-Time PCR

**Fig. 3:**
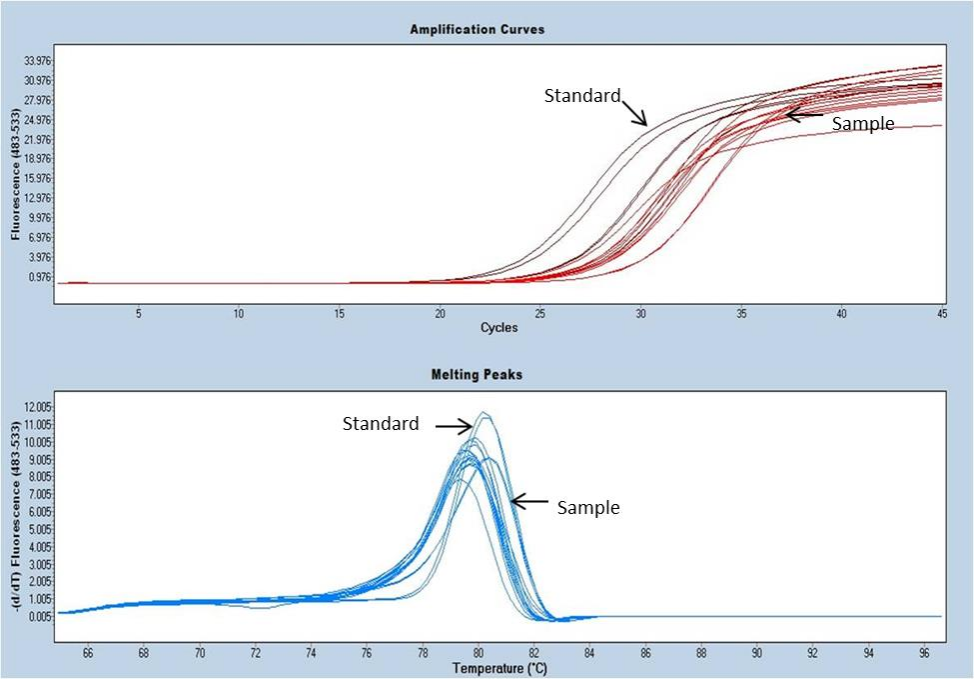
Amplification curve showing the maroon color curve for standard used for quantification and red color curve are of samples with a range of 2,68,526 DNA copies/μl-10,86,377 DNA copies/μl and melting peaks of samples in dark blue and standards in light blue, of the plasmid DNA, using genus primers Psp1 and Psp2

DNA concentrations of 5 freeze-thaw samples in SDW at −80 °C quantified using Real-Time PCR were from 2,00,000 to 11,00,000 DNA copies/μl with reference to the standard curve. Among these, the least number of copies (2,68,526 DNA copies/μl) were amplified in FP3E and highest (10,86,377 DNA copies/μl) in FP4E sample (Fig. 3).

### DNA Quantification using PCR

**Fig. 4:**
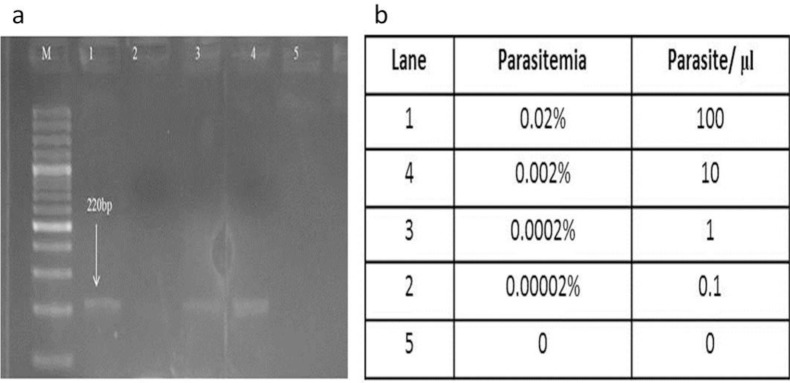
Sensitivity of freeze-thaw based detection of Plasmodium samples following 10-fold serial dilution. (a) A genus-specific fragment of the mitochondrial gene was amplified by PCR from serial dilutions genomic DNA extraction. The result following polyacrylamide gel (2.5%) electrophoresis shows that the assay’s limit of detection is approximately 1 parasite/l (lanes 3, panel b)

The freeze-thaw diluted samples were detected using mitochondrial gene. The detection limit of the samples was approximately 10 parasite/μl (Fig. 4)

### PCR Amplification

**Fig. 5:**
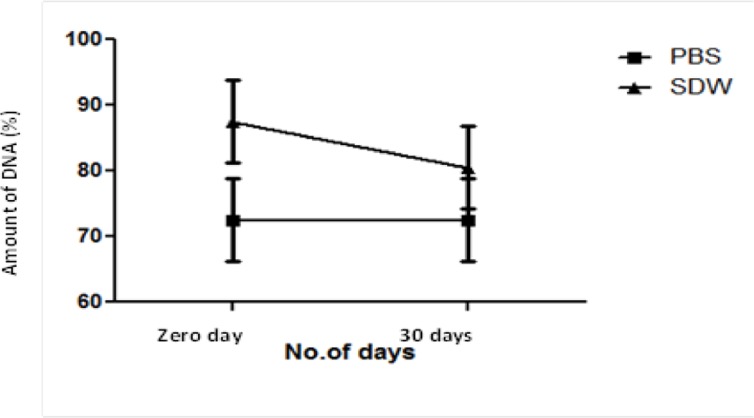
Correlation of amount of DNA in two solvents: Sterile Distilled Water (SDW) and Phosphate Buffer Saline (PBS), immediately after DNA isolation and after storage at 0 °C for 30 d

DNA samples were analyzed by amplification assays on zero-day and 30^th^ day after storage at 0 °C by targeting 18S rRNA gene of *Plasmodium*. With PBS, DNA efficiency was found to be consistent with 73% amplifications on both zero day and 30^th^ day whereas a decrease of 7% amplifications was observed for DNA stored in SDW for 30 d; with 87% amplifications on zero-day and 80% on 30^th^ day (Fig. 5).

**Fig. 6:**
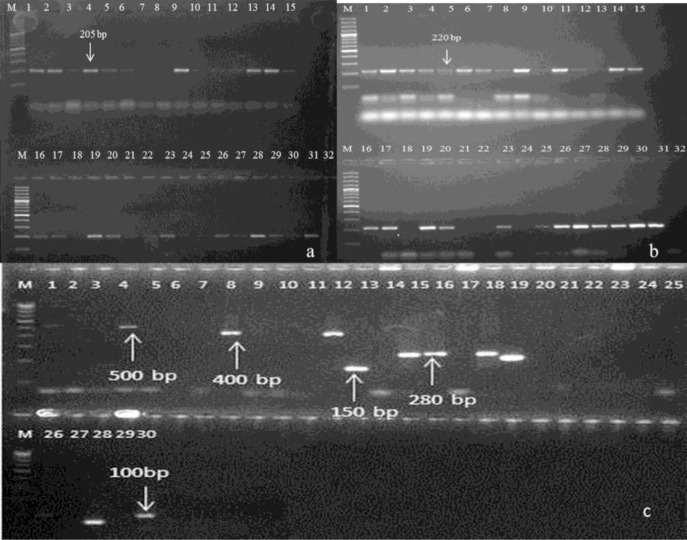
a: Amplification of 18srRNA gene of *Plasmodium* using the freeze-thaw samples. M-100 bp marker. 1-30 are samples of freeze-thaw in sterile distilled water and phosphate buffer saline at different freezing temperature (−80°C, −20°C and 0°C) respectively, 31-positive control and 32- negative control **b:** Amplification of mitochondrial gene of Plasmodium using the freeze-thaw samples M-100 bp marker. 1–30 are samples of freeze-thaw in sterile distilled water and phosphate buffer saline at different freezing temperature (−80°C, −20°C and 0°C ) respectively, 31-positive control and 32- negative control **c:** Showing the amplification of single copy gene msp1 and msp2 and its alleles using freeze-thaw samples thaw at −80°C. Lane 1 for D allele=D1, Lane 2=D2, Lane 3=D3, Lane 4=D4, Lane 5=SDW, Lane 6=D6, Lane 7 F allele= F1, Lane 8=F2, Lane 9=F3, Lane 10= F4, Lane 11=SDW, Lane 12=F5, Lane 13 for K allele=K1, Lane 14=K2, Lane 15=K3, Lane 16=K4, Lane 17=SDW, Lane 18=K5, Lane 19 for R allele=R1, Lane 20=R2, Lane 21=R3, Lane 22=R4, Lane 23=SDW, Lane 24=R5, Lane 25 for M allele=M1, Lane 26=M2, Lane 27=M3, Lane 28=M4, Lane 29=SDW, Lane 30=M5. D=IC3D7 allele, F=FC27 allele, K=K1 allele, R=R033 allele and M=MAD20 allele, M-100 bp marker

Freeze-thaw samples at −80 °C were found to be amplifiable with diagnostic genes of *Plasmodium* targeting 18S rRNA and mitochondrial gene (Fig: 6a, 6b). All 5 freeze-thaw samples at −80 °C were successfully analyzed by PCR for single copy genes *msp1*and *msp2* amplification. After the PCR assay, the classification of the alleles was done according to the number and size of fragments and the allelic family (Fig: 6c). The *msp1* gene block-1 amplification for K1 allelic family was positive for 2 samples. The other family MAD20 in *msp1*was found in 1 sample depicting allelic sizes within 100–300 bp. RO33 family was detected in 3 samples having two distinct alleles with fragment sizes of 100–200 bp. The *msp2* amplification for FC27 family was positive in 1 isolate having 280 bp fragment size. The IC3D7 allelic family was amplified in single isolates of fragment size 100bp.

### RFLP Analysis

**Fig. 7:**
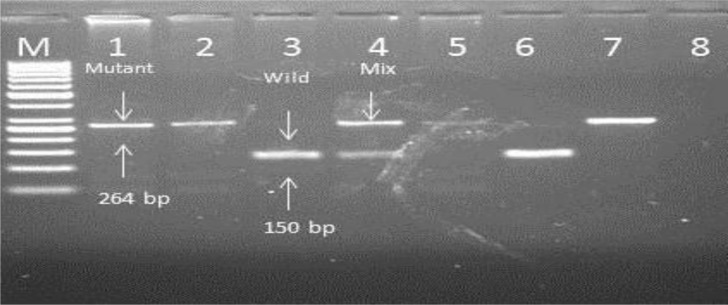
Analysis of *Pfcrt* result Lane 1=FP1E, Lane 2=FP2E, Lane 3=FP3E, Lane 4=FP4E, Lane 5=FP5E, Lane 6=MRC2, Lane 7=RKL9, Lane 8=SDW

RFLP analysis of *pfcrt* gene using ApoI restriction enzyme showed positive result. Out of five samples used in the study; RFLP pattern displayed 3 to be mutants (FP1, FP2 and FP5), FP4 being wild type and FP3 showed the presence of mixed infection with both wild type and mutant *pfcrt genes* present (Fig. 7).

### Comparison with other DNA extraction methods

The relevance of this new freeze-thaw based DNA extraction method was tested by comparing it with other known DNA isolation methods for *Plasmodium*. We found this freeze-thaw based approach best for 2 samples: FP1 and FP2, in terms of total amount of DNA extracted. For remaining 3 samples, Saponin-EDTA method produced the maximum DNA yield (Table 2). However, it needs to be considered here, that Saponin-EDTA based method is a long procedure requiring one overnight incubation step and is much expensive requiring different chemical reagents, in comparison to much cheap and rapid freeze-thaw based DNA isolation procedure.

**Table 2:** Average concentrations in ng/ μl for DNA isolated using different extraction methods

***Sample ID***	***Tris-EDTA***	***Saponin*** ***EDTA***	***Microwave***	***Freeze-Thaw***	***QIAGEN***	***BIOCHEM***
FP 1	7 2.6	1 65.3	8.5	2 07.2	3.93	1 0.35
FP 2	2 1.4	1 25.2	2 3.7	2 78	3.4	4.6
FP 3	1 03.85	1 43.3	3.1	4 2.9	1.3	6.8
FP 4	6 0	107.9	1.6	7 3.35	6.5	1 6.23
FP 5	5 4.65	1 40.7	0.2	5 9.9	2.76	4.7

### Storage stability of extracted DNA

**Fig. 8:**
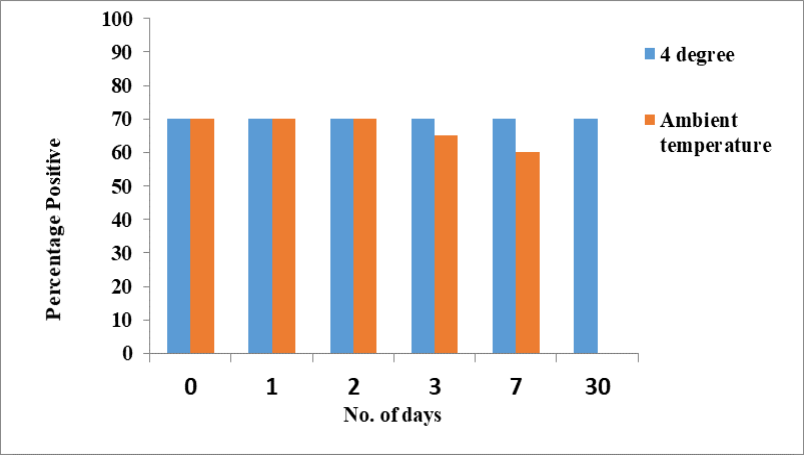
Graph showing the stability of freeze-thaw samples kept at 4 °C and ambient temperature (~28 °C). These samples were soaked in phosphate buffer saline and analyzed using amplification of mitochondrial gene on 1^st^, 2^nd^, 3^rd^, 7^th^ and 30^th^ d of storage at specific temperatures.

Storing at room temperature and 4 °C also checked the storage stability of isolated DNA. This was done by carrying out mitochondrial PCR at 1^st^, 2^nd^, 3^rd^, 7^th^ and 30^th^ d after storage; with control being the PCR done before storage (0^th^ day) (Fig. 8). Percentage of positive results remained same as the control at all tested days when the DNA samples were stored at 4 °C. With storage at room temperature, the percentage of positive results started declining at 3^rd^ day with no amplification observed at 30^th^ day.

### Sequence analysis of DNA

**Fig. 9:**
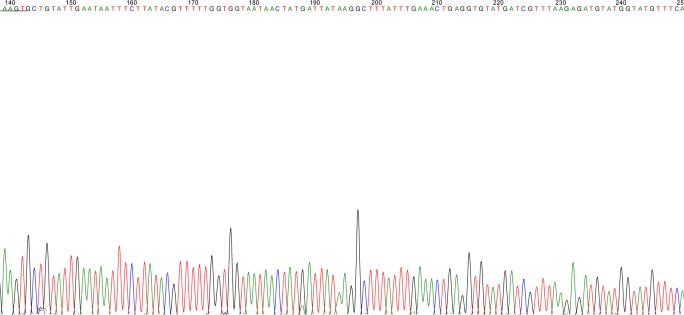
Sanger sequence analysis of the freeze-thaw samples showing sharp peaks of nucleotides

On analyzing the DNA sequence of samples, high-pitched and distinct peaks were obtained which were of high quality (Fig. 9). One of the sequences submitted for K allele got accession number KR819886 in gene bank.

## Discussion

The central role of buffers is to maintain the integrity of DNA throughout the isolation process and in future. The primary reason of using PBS in this study is that it fulfills all the requirements to be used as a chemical at field set-ups that is, it is inexpensive, non-toxic (18) and most commonly used buffer for suspending cells during rapid DNA isolation procedures (19–21). Comparing the amplification results at first day and after 30 d, consistency was observed with PBS and the amplification efficiency of DNA decreased in case of SDW.

On evaluating the data for both eluting solutions at zero-day and 30^th^ day after isolation an interesting fact surfaced. At a freezing temperature of 0 °C, initially, the DNA concentration was 86.7 ng/μl and later on after 30 d storage at 0 °C it increased to 151.78 ng/μl. This unusual increase in DNA concentration could be due to the presence of intact RBCs in sample frozen at 0 °C, which got disintegrated when the sample was again stored at 0 °C and thawed. As expected, the DNA concentrations for other freezing temperatures reduced after 30 d in both solutions.

Storage at 4 °C gave same percentage of positive results as the control at 0 and 30 d; however, with storage at room temperature, the percentage of positive results started declining at 3^rd^ day with no amplification observed at 30^th^ day. This is an expected result because room temperature is certainly not a suitable condition for storing isolated DNA.

The freeze-thaw isolated DNA was quantified by Real-Time PCR and the amount of DNA in copies/μl of sample were much higher and found to be sufficient for any gene analysis of *Plasmodium*. We showed that the freeze-thaw sample on dilution gives a significant result for limit of parasite detection which was roughly 10 parasite/μl detection. The limit of detection can further be improved when we will use 3 punches of blood spot instead of one as proposed in this study.

The quality of DNA matters a lot for sequencing of any *Plasmodium* gene because of the hindrance caused by human DNA (22), ideally it is suggested to remove human DNA by high-speed centrifugation but our DNA samples were fit for sequencing with a homology of 95%–98% nucleotide identity with NCBI BLAST nucleotide sequences (Table 3).

**Table 3:** NCBI BLAST analysis of *P. falciparum* msp-1 and msp-2 DNA genotyping results from the freeze thaw samples

***Sample***	***Allele size (bp)***	***BLASTN highest homology, GenBank description***	***E value***	***% Nucleotide identity***	***% query coverage***
D4	801	XM_001349542.1 Plasmodium falciparum 3D7 merozoite surface protein 2 precursor (MSP2) mRNA, complete cds	7e–158	96	84
F1	741	JX885915.1 - Plasmodium falciparum isolate TAB127 merozoite surface protein 2 (MSP2) gene, partial cds	6e–168	99	91
K2	401	HM568594.1- Plasmodium falciparum isolate 287 merozoite surface protein-1 (MSP-1) gene, partial cds	1e–93	98	49
M2	232	HM153228.1 Plasmodium falciparum isolate 2/044-1-2 merozoite surface protein 1 (msp1) gene, partial cds	4e–46	98	95

NCBI = National Center for Biotechnology Information; BLAST = basic local alignment search tool; msp = merozoite surface protein 1; bp = basepairs.

DNA isolated using the simple freeze-thaw approach without any purification step was found to be digestible with restriction enzymes. This suggests isolation protocol is suitable for low resources set-ups. We further explored the potential of isolated DNA for single copy gene (*msp* 1 and *msp2*), conventionally used for genetic analysis of *P. falciparum* population. These polymorphisms have been found sufficient to define any particular *P. falciparum* isolate and have potential for analysis as biological and epidemiological investigations (23).

Further, the significance of this new freeze-thaw based DNA extraction method was tested by comparing it with other known DNA isolation methods for *Plasmodium*. However, all chemical based methods are expensive and they require different chemical reagent whereas, in comparison, our freeze-thaw based DNA isolation is an inexpensive procedure.

## Conclusion

Among the many known methods for *Plasmodium* DNA isolation, our suggested method is novel as it requires only one punch of DBS for analysis, single time freezing and thawing, is chemical free, least expensive and less labour intensive compared to conventional methods for malaria parasite DNA isolation. It could be the method of choice for LAMP assay which has wide field applicability.
